# In Vitro Comparison of Device‐Induced Hemolysis, Platelet Defects, and von Willebrand Factor Degradation Between the HeartMate 2 and HeartMate 3 Pumps

**DOI:** 10.1111/aor.15013

**Published:** 2025-04-24

**Authors:** John Vandenberge, Dong Han, Wenji Sun, Shigang Wang, Douglas Tran, Nancy Kim, Kiersten Clark, Randy Perez, Bartley P. Griffith, Zhongjun J. Wu

**Affiliations:** ^1^ Artificial Organs Lab, Department of Surgery University of Maryland School of Medicine Baltimore Maryland USA; ^2^ Fischell Department of Bioengineering, A. James Clark School of Engineering University of Maryland College Park Maryland USA

**Keywords:** blood damage, heart failure, HeartMate 2, HeartMate 3, hemolysis, platelet activation, ventricular assist device

## Abstract

**Background:**

Left ventricular assist devices (LVADs) have been utilized to maintain the circulatory demands of patients with end‐stage heart failure. Despite their positive impact, hemocompatibility‐related adverse events remain a major challenge. The aim of this study is to compare in vitro hemocompatibility performance between the HeartMate 2 (HM2) and HeartMate 3 (HM3) pumps by assessing device‐induced blood damage in an in vitro circulatory loop.

**Methods:**

Fresh healthy human blood was circulated for 4 h in a circulatory loop assisted by an HM2 or HM3 pump at a flow rate of 4.5 L/min and a pressure head of 75 mmHg. Hourly blood samples were collected for analysis of hemolysis, platelet activation, platelet receptor shedding, and high molecular weight multimer (HMWM) degradation of von Willebrand factor (VWF).

**Results:**

The data from the hourly blood samples showed that the HM3 pump caused significantly lower levels of hemolysis, platelet activation, platelet receptor shedding, and HMWM degradation of VWF compared to the HM2 pump.

**Conclusion:**

The HM3 exhibited superior overall hemocompatibility to the HM2, underscoring the advantages of the fully magnetically levitated centrifugal pump design in the HM3 compared to the mechanical bearing‐supported axial pump design of the HM2.

## Graphical abstract

This in vitro comparative study demonstrated that the HeartMate 3 pump has a superior overall hemocompatibility profile to the HeartMate 2 pump, underscoring the advantages of the maglev centrifugal pump design compared to the axial pump design.

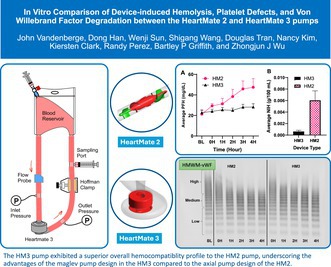

## 1 Introduction

Heart disease has been the leading cause of death in the United States since 1950, with an estimated annual cost of $30 billion to support patients who progress to heart failure (HF) [1, 2]. For patients with end‐stage HF, heart transplantation (HT) is the preferred treatment. However, not all patients meet the criteria for HT. Even for those who meet the HT criteria, they often have to wait a prolonged time due to the limited availability of donor organs [3]. Mechanical circulatory support (MCS) using left ventricular assist devices (LVADs) offers a vital alternative when HT is not a viable option. LVADs can improve the quality of life for those patients, yielding survival rates approaching HT [4, 5]. Early VADs mimicked the function of the natural human heart by employing a “pulsatile” action, alternately drawing blood from the left ventricle into the pump and ejecting it into the aorta. However, recent advances in continuous‐flow LVADs based on rotary pump technology have introduced smaller, more durable devices that outperform pulsatile VADs in terms of longevity and reliability.

The HeartMate 2 (HM2) (Abbot, Pleasanton, CA, USA), approved by the FDA in 2008, is considered the first widely approved continuous‐flow LVAD in clinical practice. It features an axial flow design supported by a pair of mechanical bearings for its impeller. However, the contact point between these bearings and the impeller, along with the high rotating speed of the impeller, can contribute to pump thrombosis that leads to device failure and severe adverse events. Other complications range from bleeding to thrombus formation, both of which can be life‐threatening [6, 7]. With advancements in magnetic levitation (maglev) technology, LVADs featuring centrifugal pumps were introduced, eliminating the need for mechanical bearings by employing magnetically supported impellers.

The HeartMate 3 (HM3) (Abbot, Pleasanton, CA, USA) represents the latest advancement in LVAD design. It features a fully magnetically levitated centrifugal pump that eliminates contact points, which helps minimize mechanical wear and reduces thrombus risk [8]. Studies have demonstrated that the HM3 offered reduced risks of stroke and pump thrombosis compared to its predecessor, the HM2 [9]. The HM3's success as a destination therapy reflected advancements in LVAD technology, making it a robust, long‐term alternative for patients who are ineligible for orthotopic heart transplantation (OHT) [10]. It has become an alternative standard of care for end‐stage HF patients who are not transplant candidates, serving as a bridge to transplant or destination therapy [11, 12, 13].

Current LVADs would inevitably generate nonphysiological shear stress (NPSS) as blood is aggressively propelled through the impeller blade framework [14]. Research has shown that NPSS can cause damage to blood elements, such as red blood cells (RBCs), platelets, and neutrophils [15, 16, 17, 18]. NPSS damages RBCs' membranes, leading to hemolysis [19, 20, 21]. If hemolysis continues unchecked, the ability of the body to clear hemoglobin will become impaired, and plasma‐free hemoglobin (PFH) may deposit in kidney glomeruli, resulting in elevated creatinine and acute kidney injury [22, 23]. Platelets are also affected by persistent NPSS, which activates the integrin complex glycoprotein (GP) IIb/IIIa, induces proteolysis of the key adhesion receptors, such as GPVI and GP1bα, leading to platelet dysfunction [15, 16, 24]. NPSS further impacts the plasma protein von Willebrand factor (VWF), essential for platelet hemostatic function. Patients with LVADs often develop an acquired VWF deficiency due to impeller‐induced shear forces that cleave the protein, degrading high molecular weight multimers (HMWM) of VWF. This, combined with platelet dysfunction, predisposes patients to bleeding complications [16, 24, 25].

The HM3, while being extensively studied in clinical settings, has not been rigorously evaluated in controlled in vitro conditions for hemocompatibility, particularly with a focus on a direct comparison to its predecessor, the HM2. In this study, the two devices were compared by assessing key indices of hemocompatibility, including hemolysis, platelet activation, platelet receptor shedding, and VWF HMWM degradation, in an LVAD‐assisted circulatory loop using human blood.

## 2 Materials and Methods

### 2.1 Blood Collection

Fresh blood was obtained from healthy adult donors through venipuncture. Each volunteer confirmed that they have not taken any anticoagulant medications or herbal supplements in the last 2 weeks prior to blood donation. The study was approved by the University of Maryland's Institutional Review Board. Informed consent was obtained in accordance with the Declaration of Helsinki. Study volunteers were informed of the goal of the study and how their blood donation would be utilized. One unit (450 mL) of whole blood was collected from the antecubital vein and stored in a sterilized blood bag (Teruflex dry collection bag, Terumo Corporation, Tokyo, Japan) containing 3.2% buffered sodium citrate solution (50 mL, Medicago AB, Uppsala, Sweden) at room temperature. Hematocrit (HCT) was adjusted to 35% ± 2% using phosphate‐buffered saline (PBS) and albumin in accordance with ASTM standards [26], achieving a total blood volume of approximately 600 mL.

### 2.2 Circulatory Loop Setup

**FIGURE 1:**
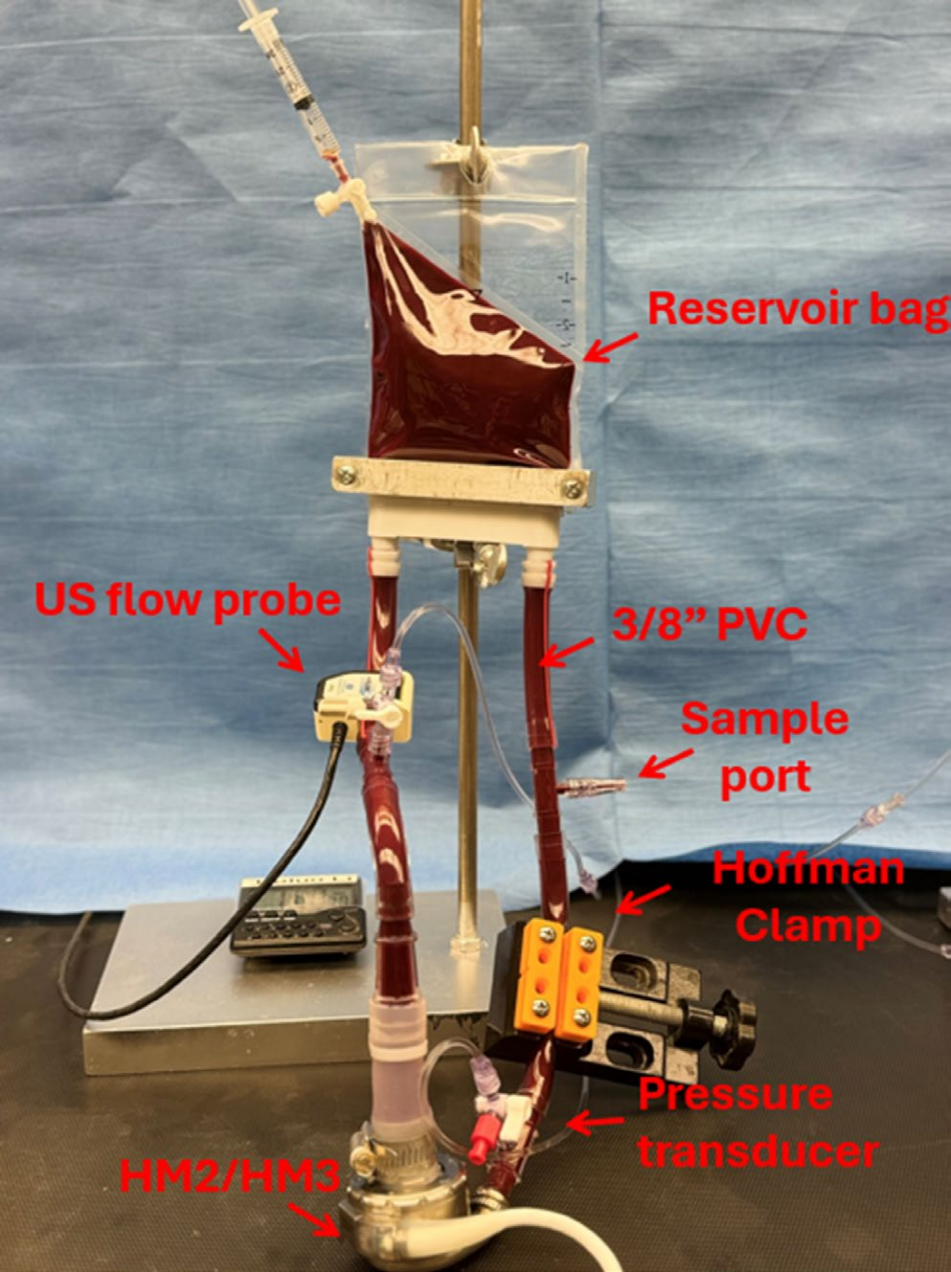
Experimental set‐up of the circulatory loop consisting of a blood reservoir, an HM2 or HM3 pump, polyvinylchloride tubing (3/8 in.), connectors, ports for blood sampling, a Hoffman clamp to adjust the pressure head, an ultrasound flow probe, and pressure transducers to measure the pressure pre and post the study pump. [Color figure can be viewed at wileyonlinelibrary.com]

The circulatory loop consists of a blood reservoir, an HM2 or HM3 pump, polyvinylchloride tubing (3/8 in.), connectors, and ports for sampling and pressure measurements (Figure 1). An ultrasonic flow probe (Transonic System Inc., Ithaca, NY, USA) was used to measure the blood volume flow rate. A Hoffman clamp was placed downstream of the blood pump to adjust the target pump pressure head. Pressure transducers (PX1800, Edwards Lifesciences) were placed pre‐ and postpump to measure pressure head. The tubing circuit was initially filled with saline to check the integrity of connections and ensure no leakage. The saline was then replaced with PBS, circulated for at least 10 min, and drained. Each circuit was filled with approximately 300 mL of blood. The rotational speed of the impeller and Hoffman clamp were adjusted to generate a flow rate of 4.5 L/min and a pressure head of approximately 75 mmHg, yielding a rotational speed of 5500 rpm (rpm) for HM3 and 10 400 rpm for HM2. A baseline (BL) sample was obtained prior to device start, and hourly blood samples (0–4 H) were obtained (5.5 mL each) once the loop was established. The 4‐h experiment was repeated at least 11 times.

### 2.3 Blood Sample Analysis

#### 2.3.1 Plasma Free Hemoglobin

One milliliter of each of the collected blood samples was centrifuged at 2000 × g for 15 min at 4°C (Thermo Electron Corp., Waltham, MA, USA). The collected plasma was recentrifuged (Fisher Scientific, Hampton, NH, USA) at 13000 × g for 15 min at 4°C. Plasma was collected for the PFH measurement. PFH concentration was determined by using a spectrophotometric assay (Sigma‐Aldrich, St. Louis, MO, USA) with a SpectraMax M3 Spectrophotometer (Molecular Devices, San Jose, CA, USA). Normalized index of hemolysis (NIH) was calculated based on the measured PFH according to the ASTM standard [26].

#### 2.3.2 Flow Cytometry for Platelet Activation and Platelet Receptor Shedding

Platelet activation and platelet surface receptor shedding were measured by flow cytometric assays. Fluorescein isothiocyanate (FITC)–labeled anti‐human PAC‐1 (BD Biosciences, San Diego, CA, USA) and PE‐labeled anti‐human CD62P (P‐Selectin, clone AK4, IgG1, κ) antibody (BioLegend, San Diego, CA, USA) were used to determine the levels of platelet surface receptor GPIIb/IIIa activation and P‐selectin expression, respectively. Platelet receptor shedding was quantified by measuring the expression levels of platelet GPIbα, GPVI, and GPIIb/IIIa. Briefly, blood samples (containing 1 × 10^6^ platelets) were added to 50 μL Tyrode's buffer (with 5 mM Gly‐Pro‐Arg‐Pro (GPRP), Sigma‐Aldrich) in 5 mL tubes and incubated with an antibody cocktail including either 20 μL PAC‐1‐FITC, 5 μL CD62P‐PE, 5 μL CD41/CD61‐PERCP, and 1 μL CD41a‐APC, or 10 μL CD42‐FITC, 1 μL GPVI‐PE, and 1 μL CD41a‐APC at room temperature in the dark for 20 min. The platelets were then fixed with 1.0 mL 1% PFA for 30 min at 4°C in the dark. The flow cytometry data of the samples were acquired on the BD Calibur (BD Biosciences, San Jose, CA, USA). More detailed information can be found in our previous publications [24, 27].

#### 2.3.3 Western Analysis of von Willebrand Factor Multimers

The loss of HMWM of VWF was assessed using Western blotting. Briefly, 4 mL blood samples were spun at 160 × g for 15 min at 20°C. The supernatant was then centrifuged at 14 000 rpm for 10 min at 4°C. Platelet‐poor plasma (PPP) was collected for analysis. Each plasma sample was mixed 1:20 with Laemmli sample buffer (BIO‐RAD, Hercules, CA, USA). Agarose gels (0.6%) were used to perform electrophoresis for separating VWF multimers at 30 mA for 30 min and then 50 mA until the tracking dye reached the bottom of the gel (~3.5 h). After electrophoresis, the protein ladders were transferred to a nitrocellulose membrane (Thermo Fisher Scientific, Waltham, MA, USA) overnight at 70 mA and 4°C. The polyclonal rabbit anti‐human VWF antibody (1:2500 dilution) (Agilent Dako, Santa Clara, CA, USA) and donkey anti‐rabbit Ig horseradish peroxidase (1:4000) were used to detect the multimers of VWF. Blots were developed with Amersham ECL western blotting detection reagent (Cytiva, Marlborough, MA, USA) and scanned with a ChemiDoc Imaging System (BIO‐RAD). Each blot contains columns of bands representing VWF multimers. The lowest molecular weight bands from 1 to 5 were classified as low, Bands 6–10 as medium, and all those bands > 10 as HMWM‐vWF. The optical density of each band was quantified with UN‐SCAN‐IT Gel 6.1 analysis software (Silk Scientific, Orem, UT, USA). The HMWM‐VWF proportion was calculated as the ratio of the total pixel intensity of HMWM‐VWF bands to the total pixel intensity of all VWF bands. The HMWM degradation of VWF in five sets of plasma samples was assessed.

### 2.4 Statistical Analysis

All data are presented as a mean ± standard error (SE). GraphPad Prism software version 9.5.1 (GraphPad Software, La Jolla, CA, USA) was used for a two‐way ANOVA statistical analysis, mixed‐effects analysis, and student *t*‐test. *p* < 0.05 were accepted as significant for all statistical tests.

## 3 Results

**FIGURE 2:**
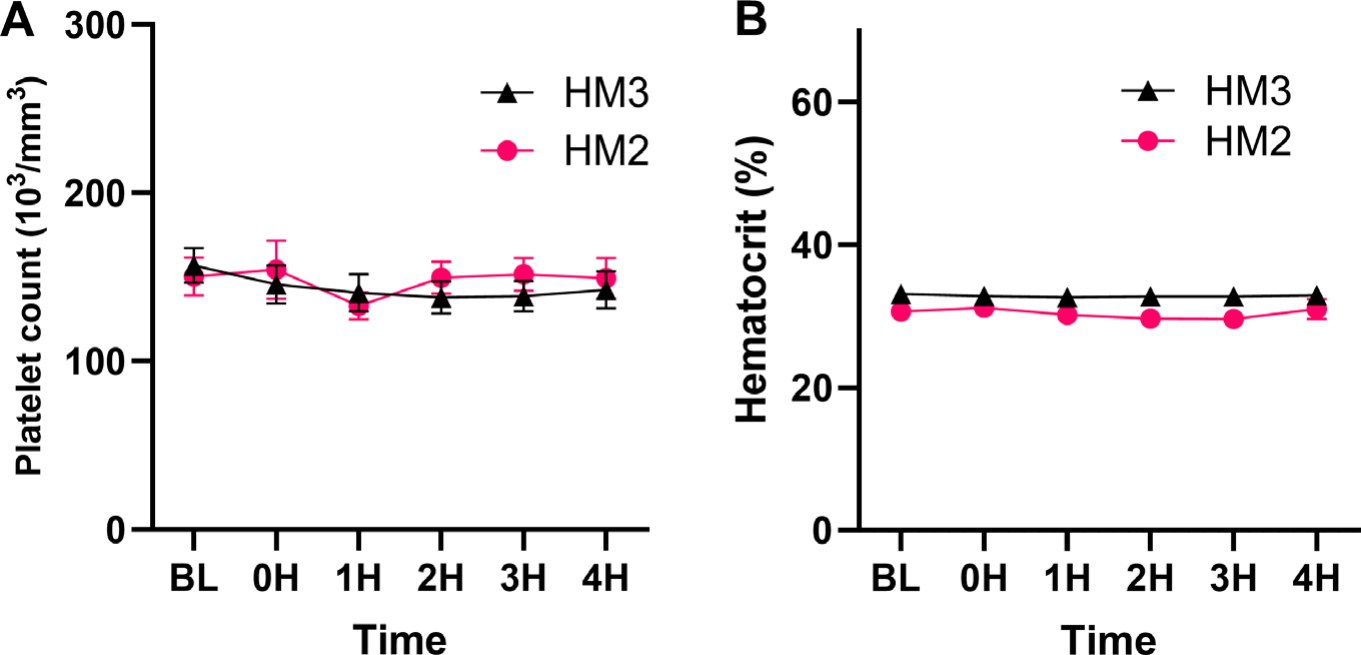
(A) Platelet count (PLT) values of the blood samples collected from the loops with the HM2 and HM3 pumps during a 4‐h circulation time. (B) Hematocrit (HCT) values of the blood samples collected from the loops with the HM2 and HM3 pumps during a 4‐h circulation time. [Color figure can be viewed at wileyonlinelibrary.com]

The platelet count and HCT in the hourly blood samples are summarized in Figure 2 for each device over the 4‐h blood circulation period. Both parameters remained stable, with no statistically significant difference observed between the baseline and hourly collected blood samples, nor between the two devices.

**FIGURE 3:**
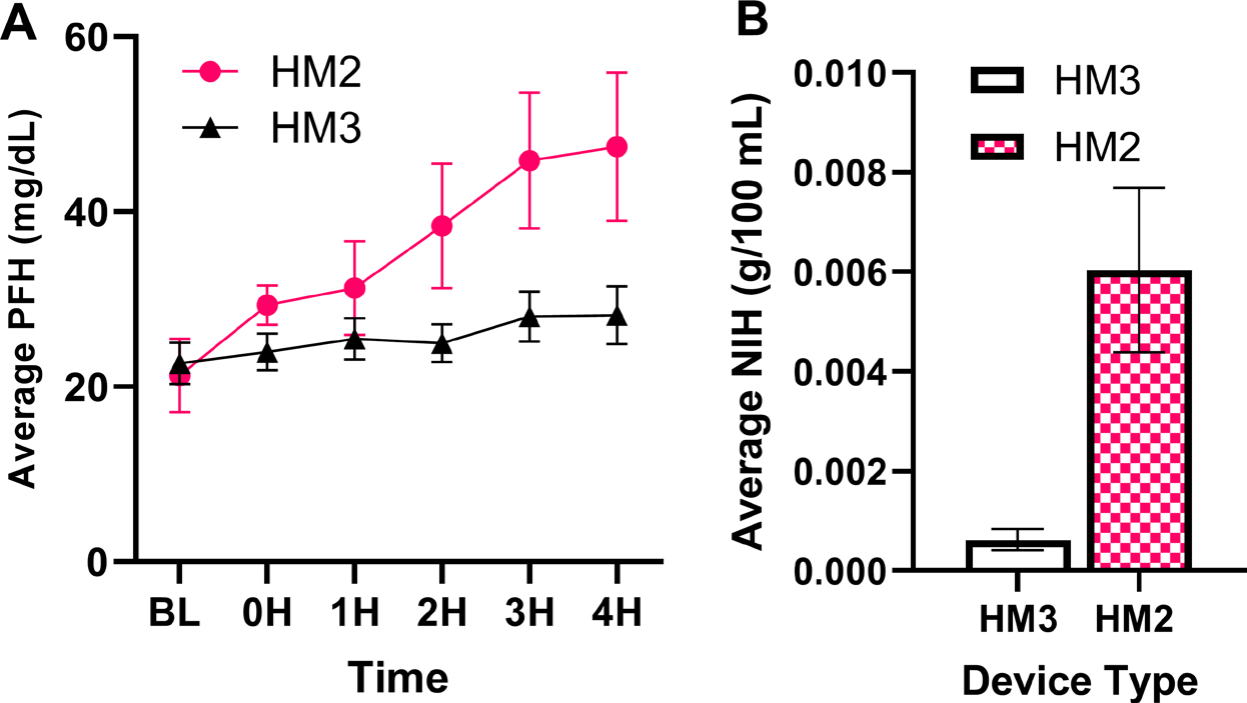
(A) Plasma‐free hemoglobin (PFH) values of the blood samples collected from the loops with the HM2 and HM3 pumps during a 4‐h circulation time. (B) Calculated average Normalized Index of Hemolysis (NIH) values for the HM2 and HM3 pumps. [Color figure can be viewed at wileyonlinelibrary.com]

The PFH concentrations in the collected blood samples exhibited an increasing trend with circulation time for both devices, as shown in Figure 3A. However, the increased rate (slope) was notably lower for the HM3 device than for the HM2 device. Consequently, the NIH value was significantly lower for the HM3 device (NIH = 0.00063 ± 0.00022 g/100 L) than for the HM2 device (NIH = 0.00604 ± 0.00165 g/100 L, *p* < 0.05), with a statistically significant different P‐value (*p* = 0.0134, *t*‐test), as illustrated in Figure 3B.

**FIGURE 4:**
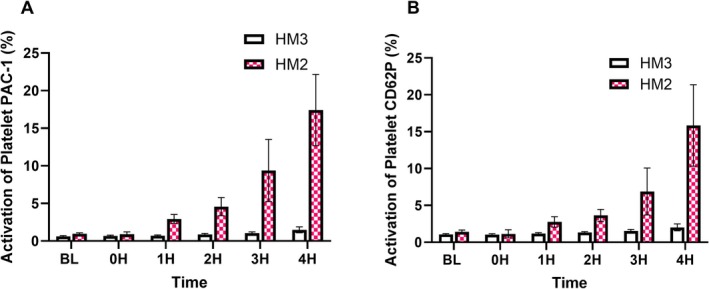
(A) Platelet activation as indicated by the activation of platelet surface receptor GPIIb/IIIa (PAC‐1) of the blood samples collected from the loops with the HM2 and HM3 pumps during a 4‐h circulation time. (B) Platelet activation as indicated by surface receptor CD62P (P‐selectin) expression of the blood samples collected from the loops with the HM2 and HM3 pumps during a 4‐h circulation time. [Color figure can be viewed at wileyonlinelibrary.com]

Platelet activation, as indicated by the biomarkers PAC‐1 (activated GPIIb/IIIa) and P‐selectin (CD62P), measured from blood samples for both HM3 and HM2 devices over time, is illustrated in Figure 4. Both PAC‐1 and P‐selectin exhibited a similar trend in the two devices, with a marked increase in HM2 after 2 h, reaching approximately 15% activation level after the 4‐h circulation. In contrast, both biomarkers remained stable in the HM3 device, with activation levels below 3% even after the 4‐h circulation. The data reveal a statistically significant difference in the levels of platelet activation between the two devices (*p* < 0.05, mixed‐effects analysis).

**FIGURE 5:**
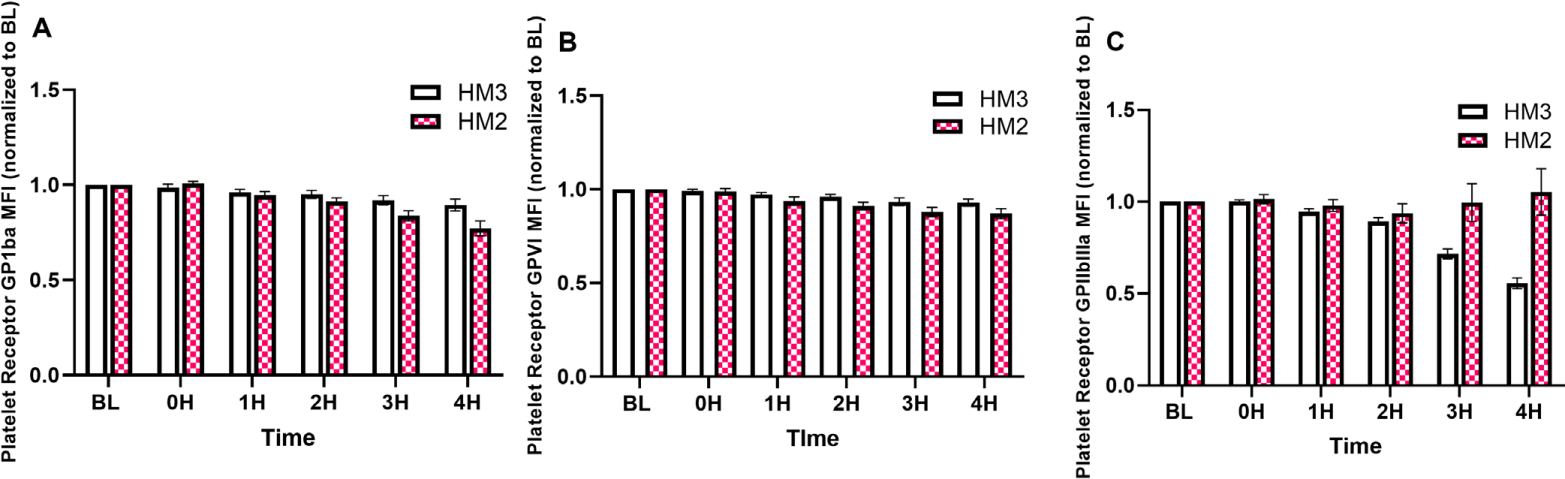
Platelet receptor shedding of (A) GP1bα, (B) GPVI, and (C) GPIIb/IIIa of the blood samples collected from the loops with the HM2 and HM3 pumps over the 4‐h circulation time. The data were represented as mean fluorescence intensity (MFI) normalized to baseline (BL) values. [Color figure can be viewed at wileyonlinelibrary.com]

The levels of platelet key receptor shedding, including GPIbα, GPVI, and GPIIb/IIIa, are summarized in Figure 5. Data are presented as normalized mean fluorescent intensity (MFI) values relative to baseline for both devices over time. GPIbα and GPVI MFI values exhibited similar trends for both devices, with a steady decrease in receptor MFI levels over time, indicating increased receptor shedding. Receptor shedding was significantly greater in HM2 than in HM3 (both *p* < 0.05, mixed‐effects analysis), with GPIbα MFI decreasing to 77% for the HM2 compared to 89% for the HM3, and GPVI MFI value decreasing to 87% for the HM2 compared to 93% for the HM3 at Hour 4. In contrast, GPIIb/IIIa MFI values showed distinct patterns between the two devices. For the HM3, a continuous decrease in MFI was observed over time, with values decreasing to 55% of baseline after 4 h of circulation. For HM2, the MFI decreased in the first 2 h, but then increased in the last 2 h, eventually reaching 105% of baseline.

**FIGURE 6:**
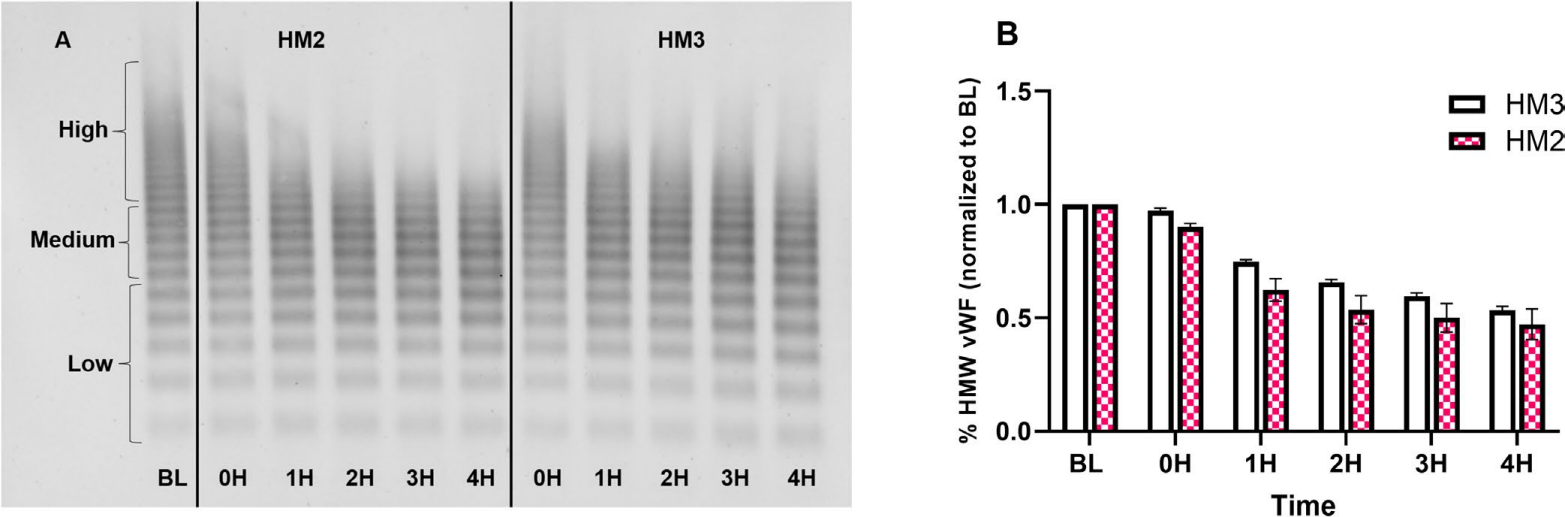
(A) Typical western blot bands of multimeric profile of von Willebrand factor of the blood samples collected from the loops with the HM2 and HM3 pumps over the 4‐h circulation time. (B) Percentage of HMWM VWF normalized to baseline (BL) of the blood samples collected from the loops with the HM2 and HM3 pumps over the 4‐h circulation time. [Color figure can be viewed at wileyonlinelibrary.com]

Figure 6A shows an image of the typical VWF multimeric profile Western blots, showing VWF bands obtained from the baseline and hourly samples for the HM3 and HM2 devices. The percentage change in HMWM‐VWF proportion, normalized to the baseline HMWM‐VWF proportion, is illustrated in Figure 6B. A steady degradation of HMWM‐VWF was observed over time for both devices. However, the VWF HMWM degradation was statistically significantly greater in the HM2 device compared to the HM3 device (*p =* 0.0258, mixed‐effects analysis), with approximately 47% of HMWM‐VWF remaining for the HM2 device and 53% remaining for the HM3 device after 4 h of circulation.

## 4 Discussion

Despite advancements in the development of LVADs, device‐related complications, such as bleeding, thrombotic events, and hemolysis‐related renal injury, remain significant challenges. Even in patients supported with the current state‐of‐the‐art HM3 LVAD, these complications are not uncommon. In this study, we compared the biocompatibility between the HM3 and its predecessor, HM2, in terms of hemolysis, platelet activation and receptor shedding, and VWF HMWM degradation using an in vitro LVAD‐assisted circulation with human donor blood. The results demonstrate that the hemocompatibility profile of the HM3 device is significantly improved compared to the HM2, with notably lower levels of hemolysis, reduced platelet activation and receptor shedding, and lower VWF degradation.

Our study showed that hemolysis in the HM3 device is significantly reduced compared to the HM2 device. Given the abundant number of RBCs and the generally lower incidence of hemolysis‐related renal injury in LVAD‐supported patients, hemolysis may no longer be a major concern in the advanced LVADs. Platelet activation was consistently lower for the HM3 device when compared to HM2. The HM3 device exhibited minimal platelet activation even after 4 h of circulation. This observation may reflect the clinical findings of reduced levels of pump thrombus formation in patients with HM3 compared to HM2. Platelet receptor shedding, as indicated by GPIbα and GPVI levels, was less pronounced in the HM3 compared to the HM2. However, GPIIb/IIIa exhibited an increased level in the HM2 at Hour 4. This difference may be attributed to the fact that, unlike GPIbα and GPVI, which have a fixed number of receptors, GPIIb/IIIa is paradoxically influenced by its upregulation during shear‐induced platelet activation due to its externalization [28, 29]. This observation aligns with the greatly increased levels of platelet PAC‐1 activation in the HM2. Therefore, the upregulation of GPIIb/IIIa is not indicative of reduced platelet impact in the HM2; rather, it signifies a greater impact compared to the HM3.

Although the HM3 device exhibited less pronounced degradation compared to the HM2, substantial VWF degradation was still observed after 4 h of circulation. As VWF plays a critical role in platelet activation and adhesion by binding its A1 domain to platelet GPIbα, degraded VWF exhibits reduced affinity for platelets, thereby impairing normal hemostatic function in patients. Acquired VWF deficiency syndrome in LVAD patients remains a significant clinical challenge, increasing bleeding risk and the likelihood of complications.

The MOMENTUM 3 trial, as reported by Mehra et al., showed that patients receiving the HM3 experienced a significantly higher event‐free 2‐year survival rate of 54% compared to the axial‐flow HM2 LVAD group, consistent with our observation that HM3 has improved biocompatibility [9]. However, the trial also highlighted that following HM3 implantation, there was a continued stroke and bleeding risk for women younger than 65 years of age over the 2‐year follow‐up period [9]. The ELEVATE registry considered the clinical outcomes of 463 patients who received the HM3 as a primary implant. At 5‐year follow‐up, the rate of overall survival free from stroke, pump thrombus, and nonsurgical bleeding was 43.8%. This implies that over 50% of patients still experienced a high risk of thrombogenic or bleeding complications within the 5‐year period [30].

This study used blood from a cohort of healthy donors, which may not adequately reflect the biochemical profile and physiology of patients with end‐stage HF. Although the devices were thoroughly cleaned after each use, only one of each device was tested. The extensive surface area of foreign materials, including tubing and reservoir bag, may have inadvertently increased blood cell interaction with nonbiological surfaces, potentially leading to cellular activation. Furthermore, the blood volume in the experimental circuit was substantially lower than that in the human body, which could exacerbate time‐dependent effects on blood damage.

## 5 Conclusions

In this study, the biocompatibility performance of the HM3 pump was assessed and compared with its predecessor, the HM2 pump, in an LVAD‐assisted circulation in vitro using human blood. Key biocompatibility parameters, including hemolysis, platelet activation and receptor shedding, and VWF degradation, were measured from collected blood samples. The HM3 exhibited superior biocompatibility, as evidenced by significantly reduced levels of hemolysis, platelet activation, platelet receptor shedding, and VWF degradation.

## Author Contributions

John Vandenberg: Data collection and analysis, manuscript writing. Dong Han: Data collection and analysis, manuscript writing. Wenji Sun: Data collection and analysis, manuscript reviewing and editing. Shigang Wang: Data collection analysis, manuscript reviewing and editing. Douglas Tran: Data collection and analysis, manuscript reviewing and editing. Nancy Kim: Data collection and analysis, manuscript reviewing and editing. Kiersten Clark: Data collection and analysis, manuscript reviewing and editing. Randy Perez: Data collection and analysis, manuscript reviewing and editing. Bartley P. Griffith: Securing funding, manuscript reviewing and editing. Zhongjun J. Wu: Securing funding, conceptualization, manuscript writing.

## Conflicts of Interest

The authors declare no conflicts of interest.
